# Supplementary material for: Characterization of Spinal Sensorimotor Network Using Transcutaneous Spinal Stimulation during Voluntary Movement Preparation and Performance
Source: J Clin Med. 2021 Dec 18;10(24):5958. doi: 10.3390/jcm10245958 (PMC8709482; doi:10.3390/jcm10245958)
Supplement: Supplementary file 1 [file jcm-10-05958-s001.zip › jcm-1478051-supplementary.pdf]

Data S1: Modulation of evoked responses during the auditory condition

This section contains the first (R1) and second (R2) responses for each of the four movement tasks during the auditory condition. R1 responses are scaled to the mean of the control data, where 1 is 100% of control. R2 responses are scaled to ratio of the R2 response to the corresponding R1 response, for that reason the control ratio is shown for comparison.

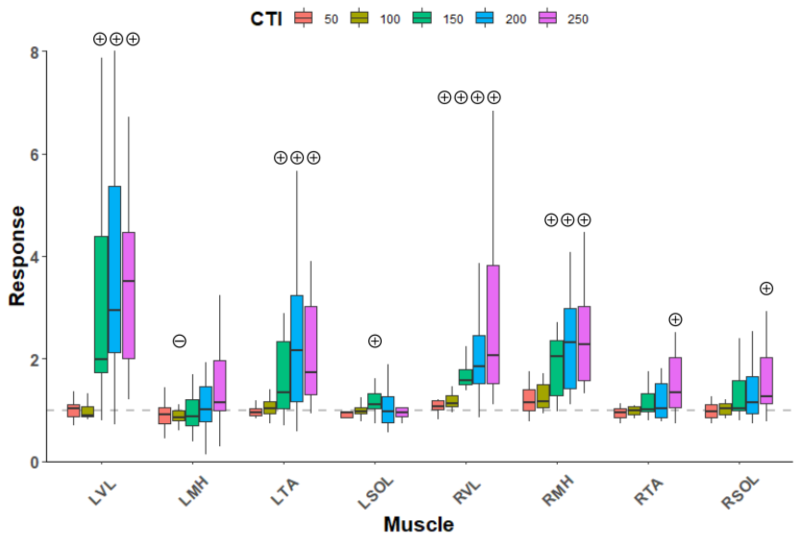

Figure S1. First responses during left knee extension using an auditory cue: A dashed line indicates the 100% control response. Significant facilitation is denoted with a circle plus symbol and significant inhibition is denoted with a circle minus. Data are grouped by muscle.

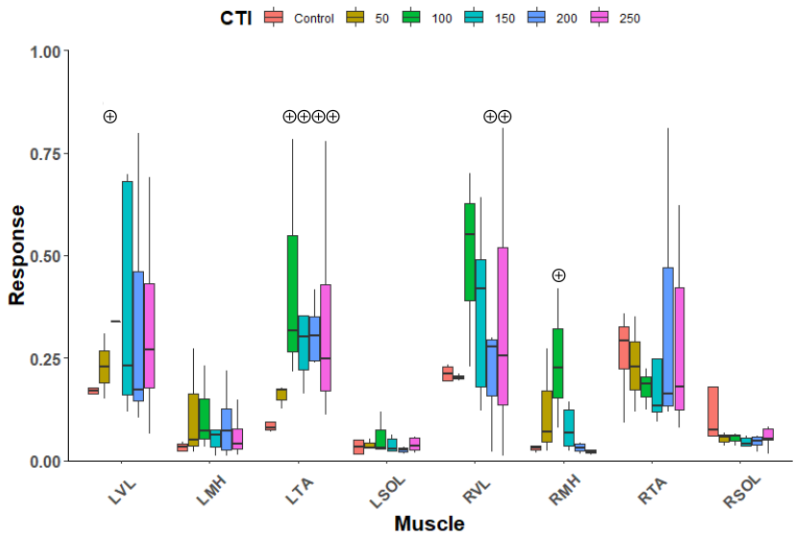

Figure S1. Second responses during left knee extension using an auditory cue: The R2/R1 ratio for control is designated as control. Significant facilitation is denoted with a circle plus symbol and significant inhibition is denoted with a circle minus. Data are grouped by muscle.

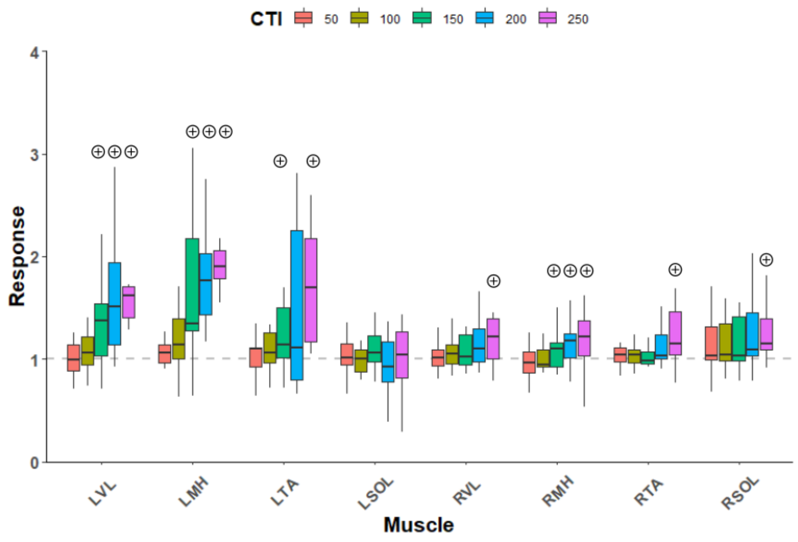

Figure S2. First responses during left knee flexion using an auditory cue: A dashed line indicates the 100% control response. Significant facilitation is denoted with a circle plus symbol and significant inhibition is denoted with a circle minus. Data are grouped by muscle.

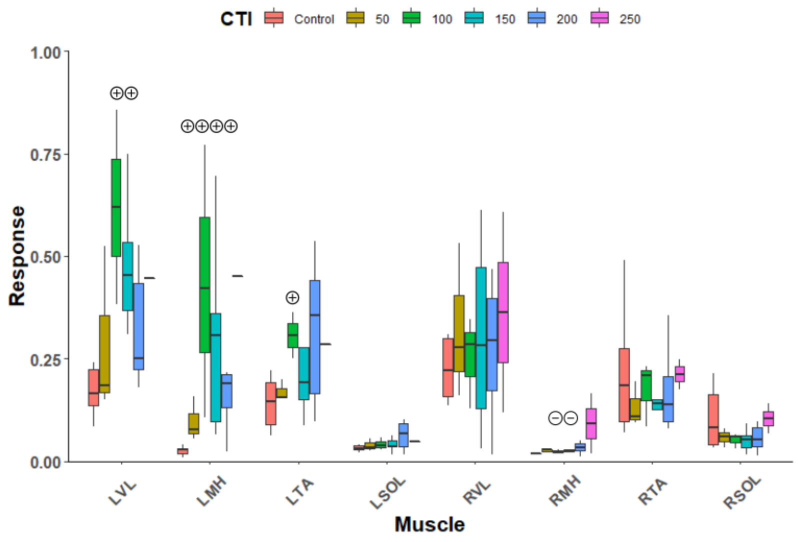

Figure S3. Second responses during left knee flexion using an auditory cue: The R2/R1 ratio for control is designated as control. Significant facilitation is denoted with a circle plus symbol and significant inhibition is denoted with a circle minus. Data are grouped by muscle.

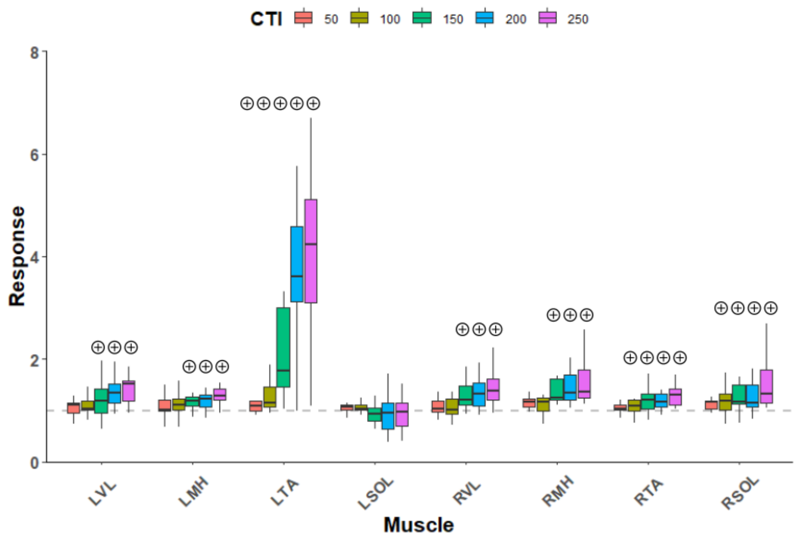

Figure S4. First responses during left dorsiflexion using an auditory cue: A dashed line indicates the 100% control response. Significant facilitation is denoted with a circle plus symbol and significant inhibition is denoted with a circle minus. Data are grouped by muscle.

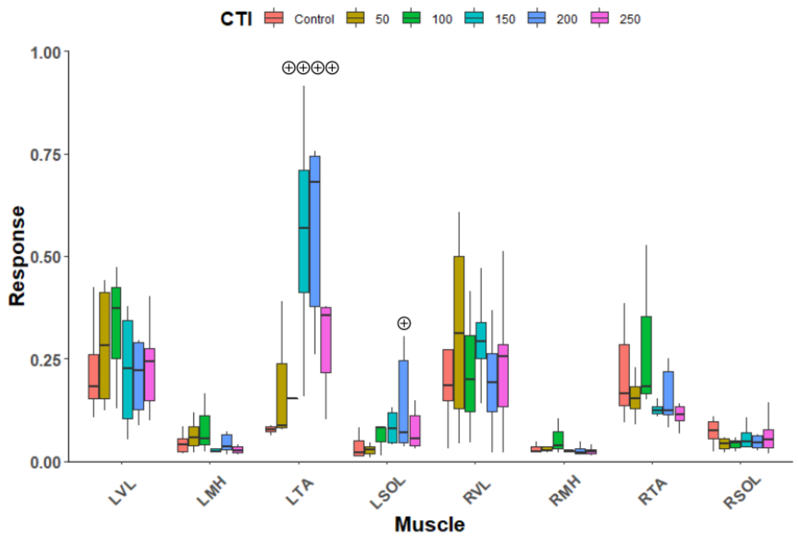

Figure S5. Second responses during left dorsiflexion using an auditory cue: The R2/R1 ratio for control is designated as control. Significant facilitation is denoted with a circle plus symbol and significant inhibition is denoted with a circle minus. Data are grouped by muscle.

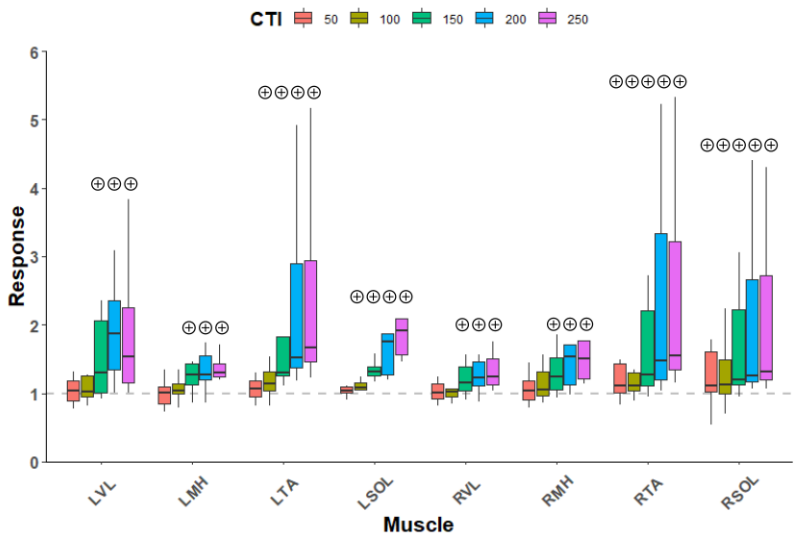

Figure S6. First responses during left plantarflexion using an auditory cue: A dashed line indicates the 100% control response. Significant facilitation is denoted with a circle plus symbol and significant inhibition is denoted with a circle minus. Data are grouped by muscle.

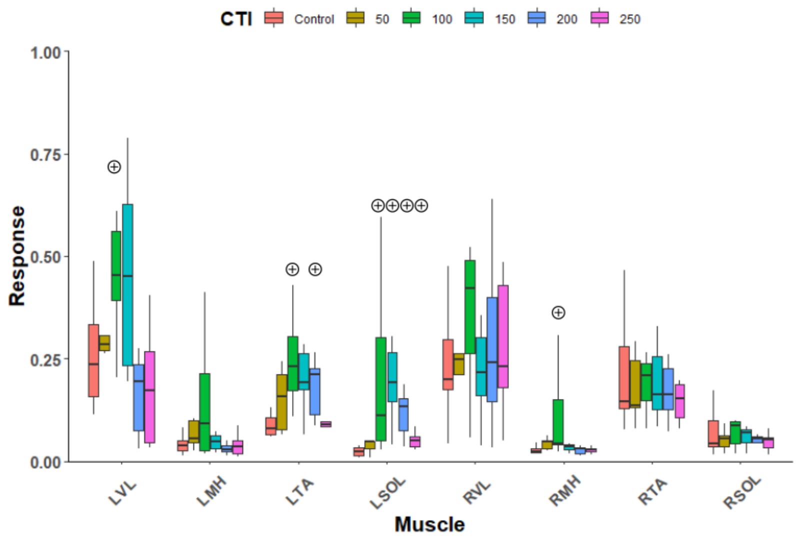

Figure S7. Second responses during left plantarflexion using an auditory cue: The R2/R1 ratio for control is designated as control. Significant facilitation is denoted with a circle plus symbol and significant inhibition is denoted with a circle minus. Data are grouped by muscle.

Data S2: Modulation of evoked responses during the tactile condition

This section contains the first (R1) and second (R2) responses for each of the four movement tasks during the tactile condition. R1 responses are scaled to the mean of the control data, where 1 is 100% of control. R2 responses are scaled to ratio of the R2 response to the corresponding R1 response, for that reason the control ratio is shown for comparison.

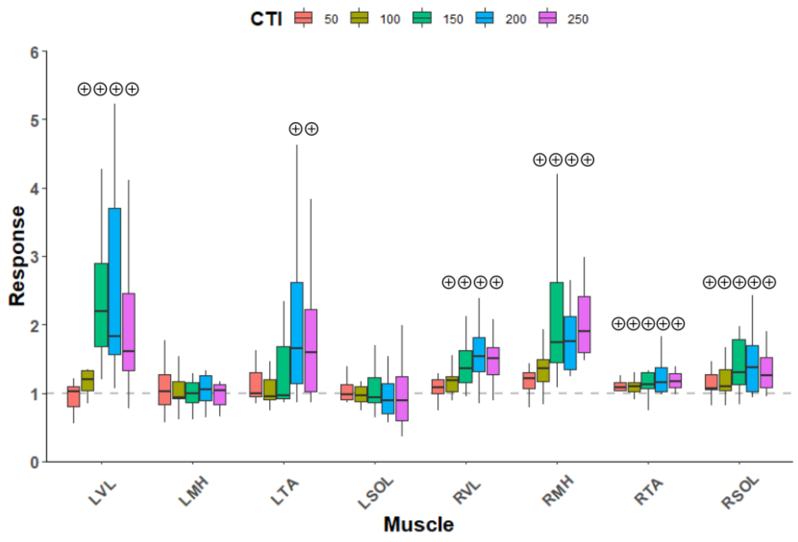

Figure S8. First responses during left knee extension using a tactile cue: A dashed line indicates the 100% control response. Significant facilitation is denoted with a circle plus symbol and significant inhibition is denoted with a circle minus. Data are grouped by muscle.

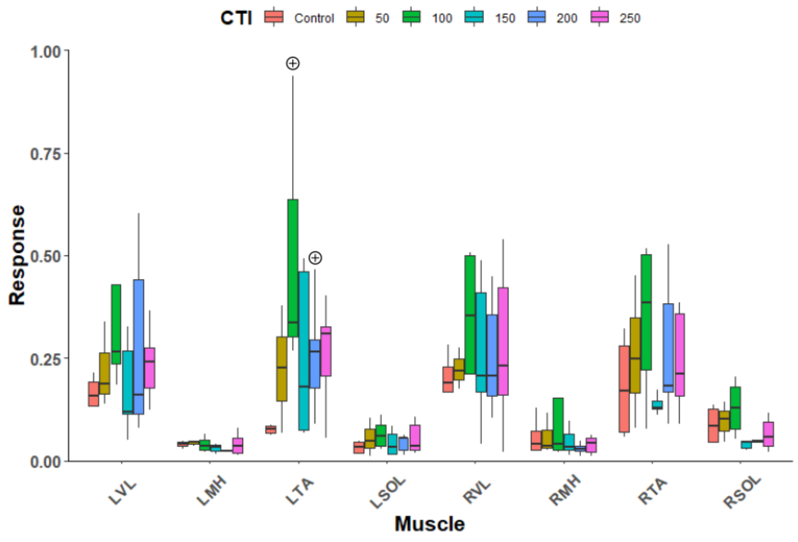

Figure S9. Second responses during left knee extension using a tactile cue: The R2/R1 ratio for control is designated as control. Significant facilitation is denoted with a circle plus symbol and significant inhibition is denoted with a circle minus. Data are grouped by muscle.

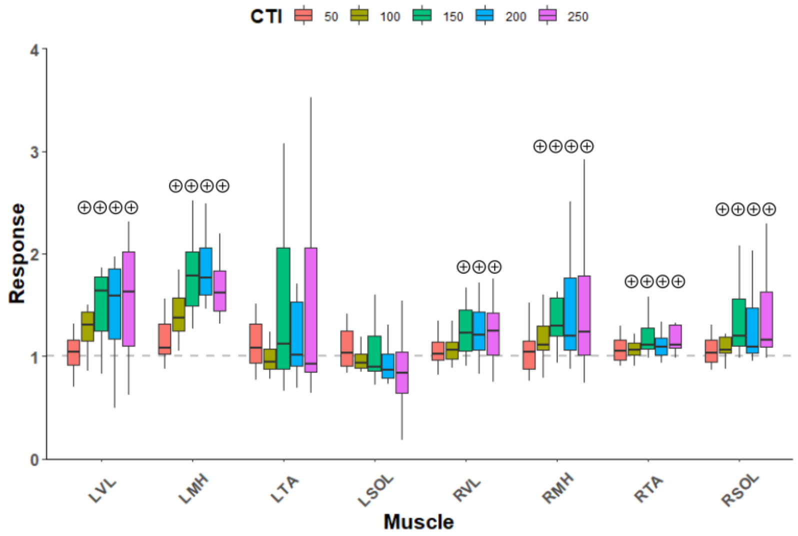

Figure S10. First responses during left knee flexion using a tactile cue: A dashed line indicates the 100% control response. Significant facilitation is denoted with a circle plus symbol and significant inhibition is denoted with a circle minus. Data are grouped by muscle.

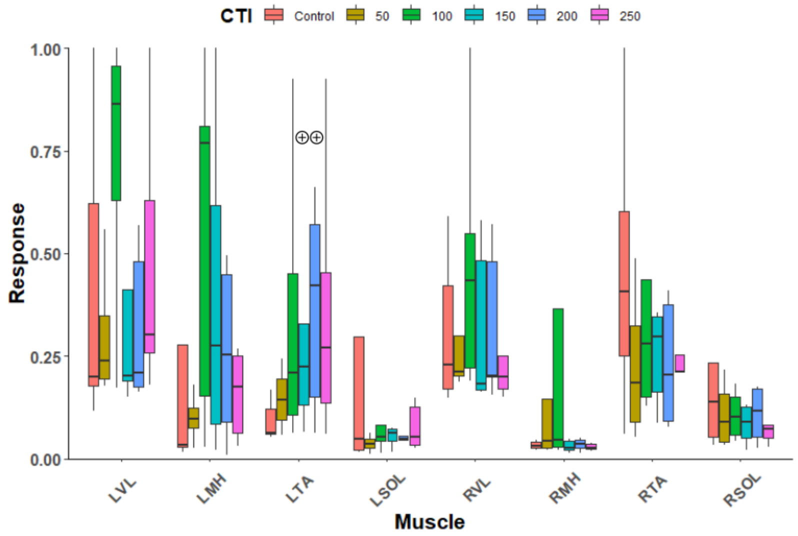

Figure S11. Second responses during left knee flexion using a tactile cue: The R2/R1 ratio for control is designated as control. Significant facilitation is denoted with a circle plus symbol and significant inhibition is denoted with a circle minus. Data are grouped by muscle.

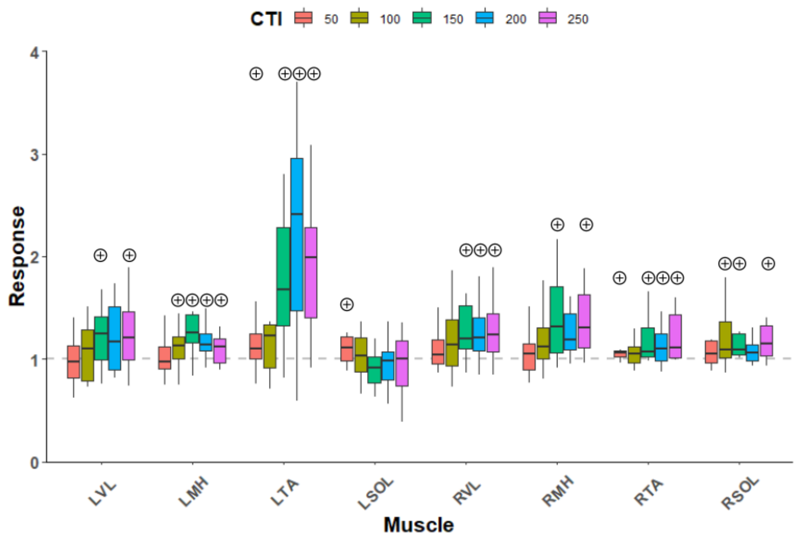

Figure S12. First responses during left dorsiflexion using a tactile cue: A dashed line indicates the 100% control response. Significant facilitation is denoted with a circle plus symbol and significant inhibition is denoted with a circle minus. Data are grouped by muscle.

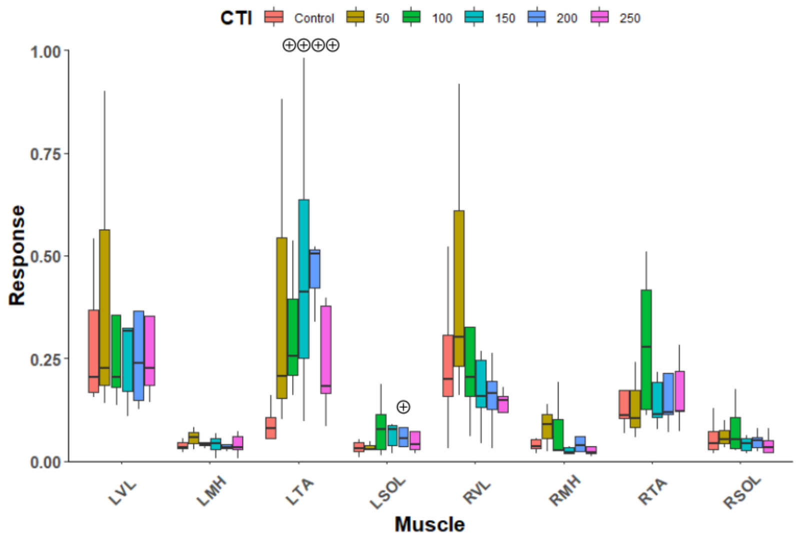

Figure S13. Second responses during left dorsiflexion using a tactile cue: The R2/R1 ratio for control is designated as control. Significant facilitation is denoted with a circle plus symbol and significant inhibition is denoted with a circle minus. Data are grouped by muscle.

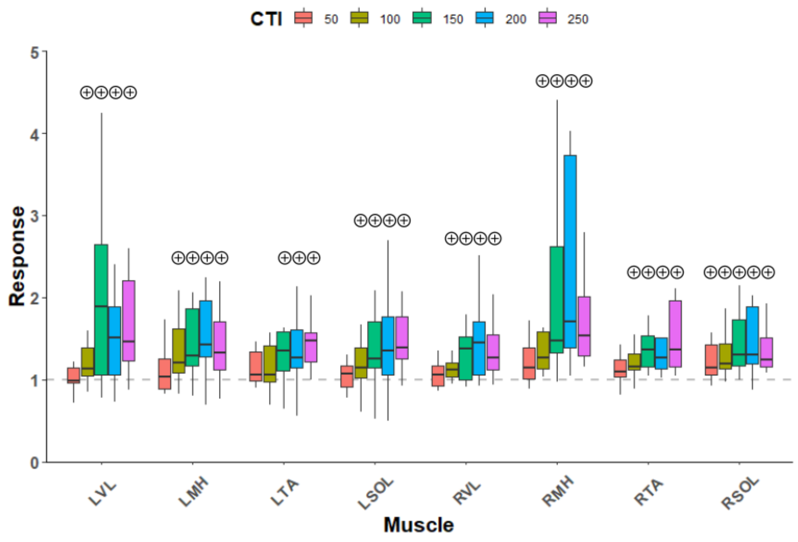

Figure S14. First responses during left plantarflexion using a tactile cue: A dashed line indicates the 100% control response. Significant facilitation is denoted with a circle plus symbol and significant inhibition is denoted with a circle minus. Data are grouped by muscle.

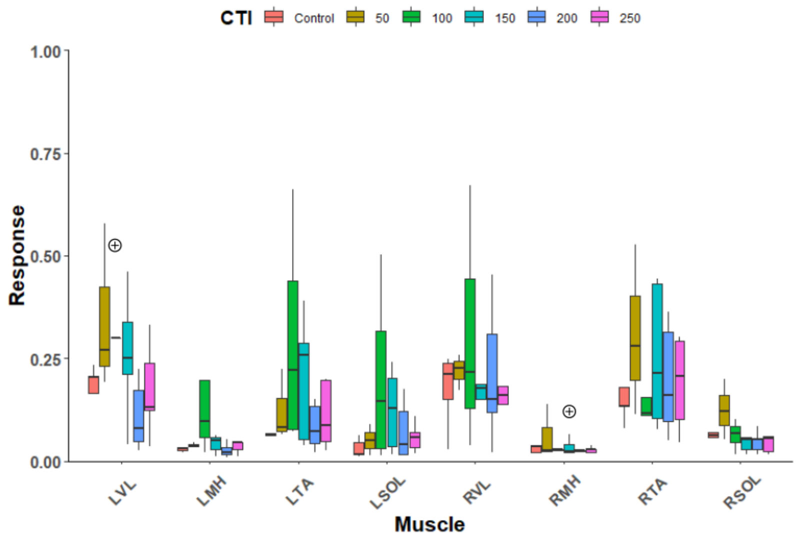

Figure S15. Second responses during left plantarflexion using a tactile cue: The R2/R1 ratio for control is designated as control. Significant facilitation is denoted with a circle plus symbol and significant inhibition is denoted with a circle minus. Data are grouped by muscle.

Data S3: Modulation of evoked responses during the isometric condition.

This section contains the first (R1) and second (R2) responses for each of the four movement tasks during the isometric contraction condition. R1 responses are scaled to the mean of the control data, where 1 is 100% of control. R2 responses are scaled to ratio of the R2 response to the corresponding R1 response, for that reason the control ratio is shown for comparison.

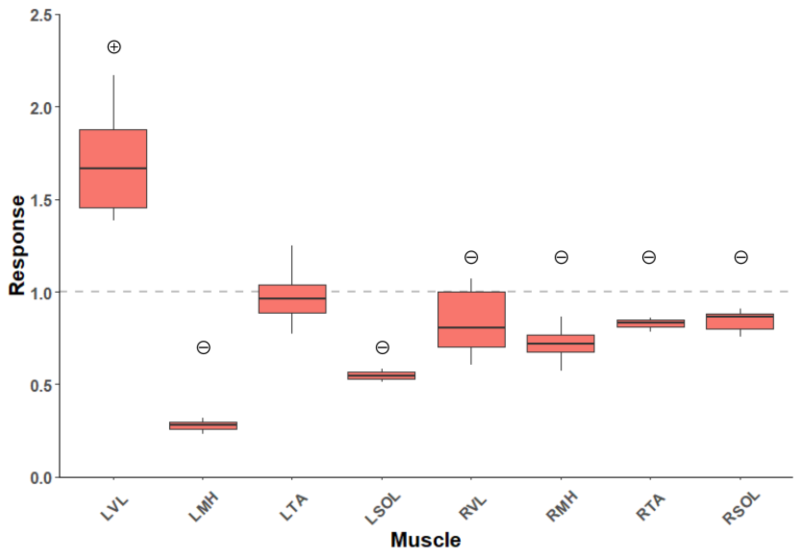

Figure S16. First responses during the isometric left knee extension task: A dashed line indicates the 100% control response. Significant facilitation is denoted with a circle plus symbol and significant inhibition is denoted with a circle minus. Data are grouped by muscle.

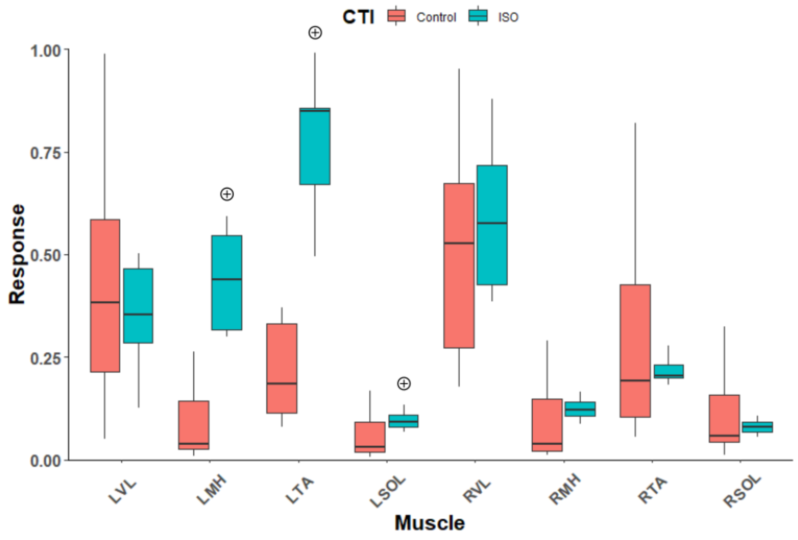

Figure S17. Second responses during the isometric left knee extension task: The R2/R1 ratio for control is designated as control. Significant facilitation is denoted with a circle plus symbol and significant inhibition is denoted with a circle minus. Data are grouped by muscle.

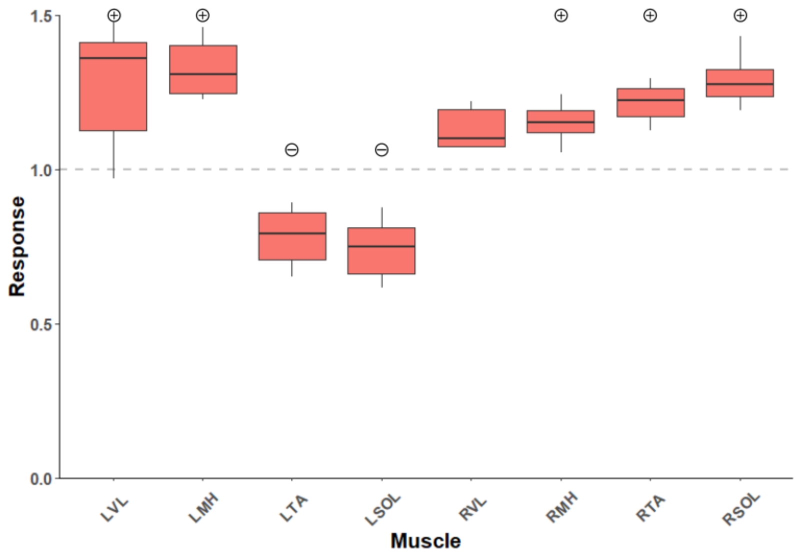

Figure S18. First responses during the isometric left knee flexion task: A dashed line indicates the 100% control response. Significant facilitation is denoted with a circle plus symbol and significant inhibition is denoted with a circle minus. Data are grouped by muscle.

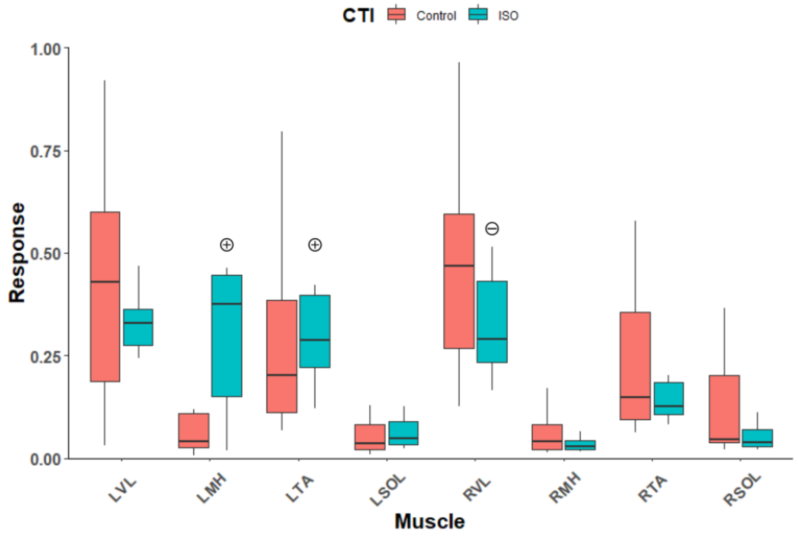

Figure S19. Second responses during the isometric left knee flexion task: The R2/R1 ratio for control is designated as control. Significant facilitation is denoted with a circle plus symbol and significant inhibition is denoted with a circle minus. Data are grouped by muscle.

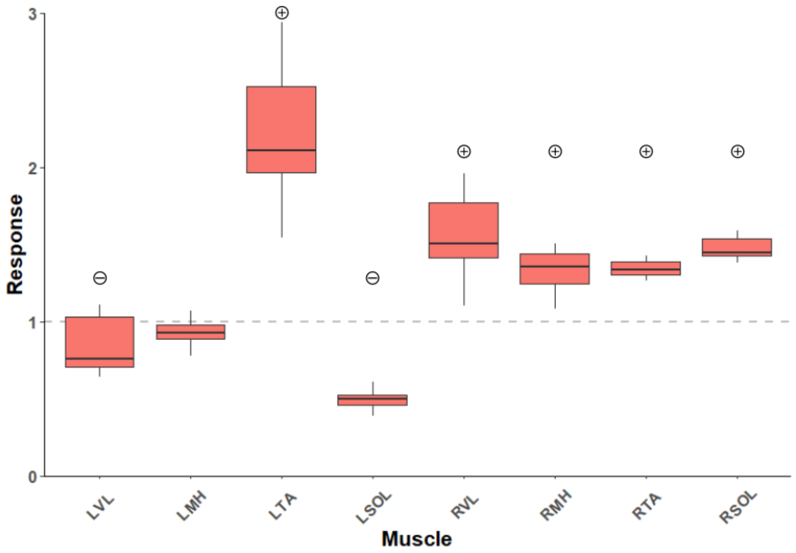

Figure S20. First responses during the isometric left dorsiflexion task: A dashed line indicates the 100% control response. Significant facilitation is denoted with a circle plus symbol and significant inhibition is denoted with a circle minus. Data are grouped by muscle.

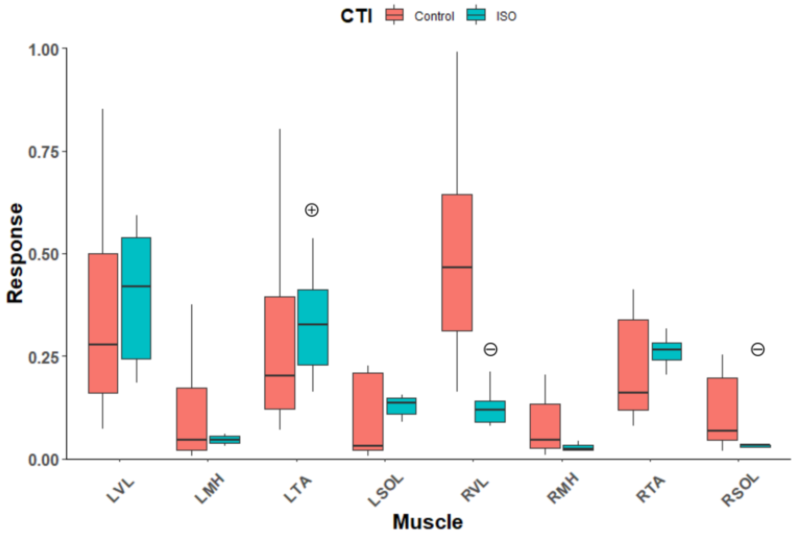

Figure S21. Second responses during the isometric left dorsiflexion task: The R2/R1 ratio for control is designated as control. Significant facilitation is denoted with a circle plus symbol and significant inhibition is denoted with a circle minus. Data are grouped by muscle.

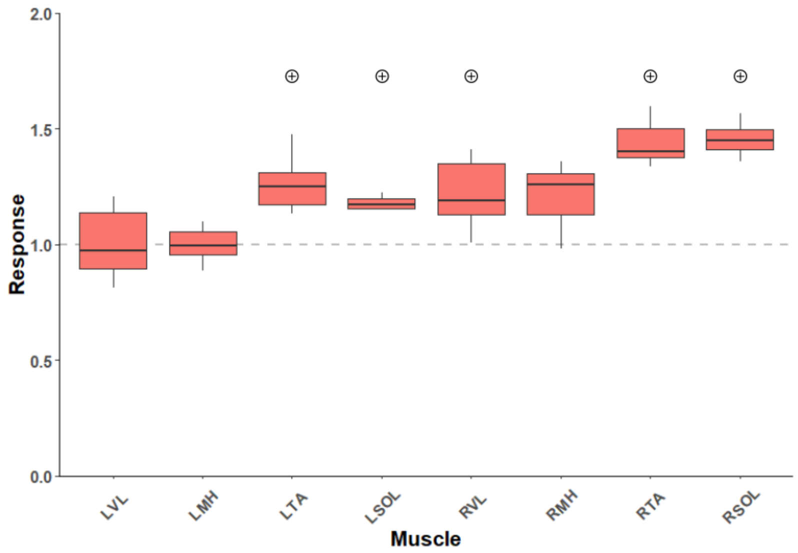

Figure S22. First responses during the isometric left plantarflexion task: A dashed line indicates the 100% control response. Significant facilitation is denoted with a circle plus symbol and significant inhibition is denoted with a circle minus. Data are grouped by muscle.

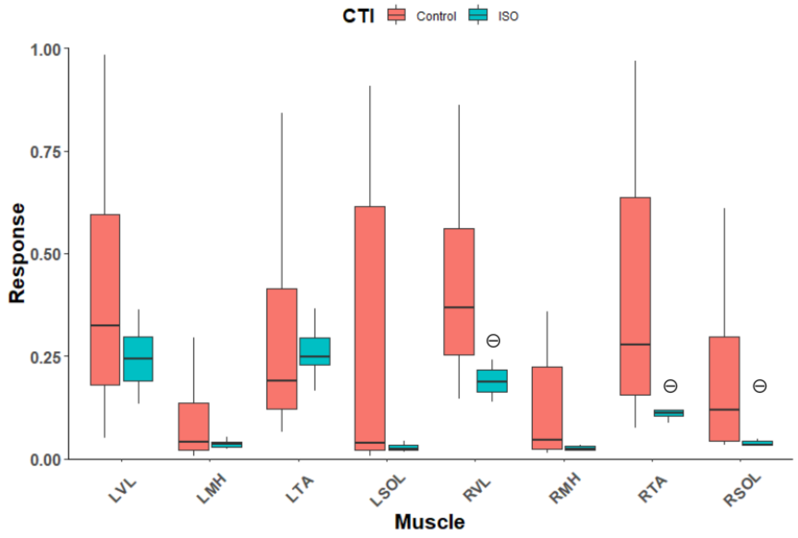

Figure S23. Second responses during the isometric left plantarflexion task: The R2/R1 ratio for control is designated as control. Significant facilitation is denoted with a circle plus symbol and significant inhibition is denoted with a circle minus. Data are grouped by muscle.
